# Long-term control of refractory acrodermatitis continua of Hallopeau with subcutaneous spesolimab: A case report

**DOI:** 10.1016/j.jdcr.2025.08.043

**Published:** 2025-10-08

**Authors:** Jan Nicolai Wagner, Franziska Zirkenbach, Brigtte Stephan, Carolin Grote, Matthias Augustin

**Affiliations:** Institute for Health Services Research in Dermatology and Nursing (IVDP), University Medical Center Hamburg-Eppendorf, Hamburg

**Keywords:** acrodermatitis continua of Hallopeau, biologics, IL-36, M. Hallopeau, spesolimab

## Introduction

Acrodermatitis continua of Hallopeau (ACH) is a rare and challenging dermatological condition, characterized by recurrent sterile pustules, typically involving the digits.^1^ ACH often results in nail dystrophy and can progress to osteitis if inadequately treated. The treatment represents a clinical challenge, as there is a lack of approved drugs and of standards by treatment guidelines.^2^ Therapies such as methotrexate, cyclosporine, and acitretin often prove inadequate in providing sufficient relief. Increasingly, biologics targeting tumor necrosis factor-α, interleukin (IL)-17, IL-23, and IL-12/23, as well as small molecules, have been reported in case series. In response to the urgent need for more effective options, spesolimab, an IL-36 receptor antagonist, has gained attention.^3^, ^4^, ^5^

## Case report

**Fig 1:**
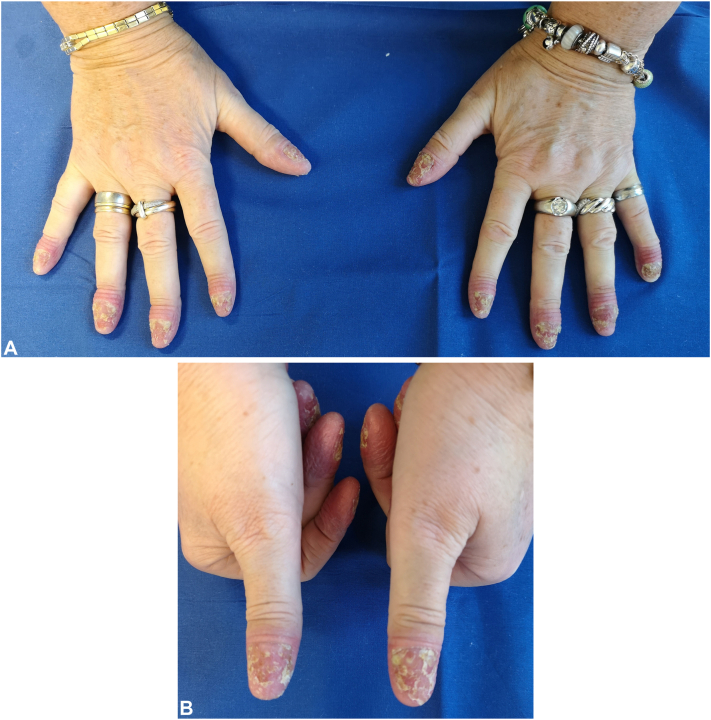
**A,** Patient’s hands: acrodermatitis continua of Hallopeau before starting spesolimab therapy. **B,** Patient’s thumbs: acrodermatitis continua of Hallopeau before starting spesolimab therapy.

A 59-year-old female patient presented in our outpatient university clinic in 2023 with a severe refractory form of ACH, first diagnosed in 2014. The condition commenced with the formation of a minor subungual pustule located beneath the left thumb. Subsequently, pustular erythematous lesions emerged on both soles. Approximately 1.5 years later, there was extensive involvement of all fingernails and swelling of distal interphalangeal joints. At the first consultation, the patient showed a drumstick-like swelling of all fingertips, with the nail structures completely absent (Fig 1, *A* and *B*).

Diagnostic workup included a biopsy in 2025 showing pustular psoriasis. A full oncological screening (mammography, colonoscopy, chest x-ray, and labs) was unremarkable. Multiple magnetic resonance imaging scans and conventional radiographs (X-rays) were performed. Imaging showed mild fasciitis and fibroostitis at the plantar fascia insertion bilaterally, evolving plantar enthesitis, and minimal erosive changes.

The patient underwent 20 systemic treatments, including conventional and biologic agents (Table I). These therapies were largely ineffective on skin lesions or limited by paradoxical reactions or side effects. The most recent treatment with bimekizumab resulted only in a partial improvement.

**Table I:** Systemic drug response and DLQI outcomes from 2014 to 2025

Year	Systemic drug	Dosage	Response	Side effects	DLQI
01/2014-06/2014	Acitretin	30 mg daily	No response		NA
06/2014-12/2014	Fumaric acid ester	120 mg daily	No response		NA
01/2015-11/2015	Methotrexate	25 mg s.c. once a wk	No response	Dyspnea, cough	NA
11/2015-12/2016	Ustekinumab	Induction: 45 mg s.c. wk 0 and 4 Maintenance: 45 mg s.c. every 12 wk	No response	Worsening of arthralgia	NA
01/2016	Systemic PUVA		Worsening of ACH		NA
12/2016	Prednisolone	20 mg daily, tapered down	Partial response	Oral candidiasis	NA
01/2017-05/2017	Secukinumab	Induction: 300 mg s.c. wk 0, 1, 2, 3, and 4 Maintenance: 300 mg s.c. every 4 wk	No response		8
05/2017-02/2018	Adalimumab	Induction: 80 mg s.c. wk 0 Maintenance: 40 mg s.c. wk 1, then every 2 wk	No response	Paradoxical psoriasis	12
02/2018-05/2018	Ixekizumab	Induction: 160 mg s.c. wk 0, 80 mg wk 2, 4, 6, 8, 10, and 12 Maintenance: 80 mg s.c. every 4 wk	Worsening of ACH		14
05/2018-08/2018	Guselkumab	Induction: 100 mg s.c. wk 0 and 4 Maintenance: 100 mg s.c. every 8 wk	No response		9
08/2018-12/2018	Apremilast	30 mg twice a d	No response	Diarrhea	14
12/2018-01/2019	Anakinra	100 mg s.c. daily	No response	Paradoxical psoriasis Oral candidiasis	14
01/2019-02/2019	Methotrexate	10 mg s.c. once a wk	No response		14
02/2019-04/2019	Infliximab	5 mg/kg BW i.v. in wk 0 and 2	No response		8
05/2019-06/2019	Tofacitinib and Risankizumab	5 mg twice a d and 150 mg s.c. in wk 0 and 4 and every 12 wk	No response		8
06/2019-08/2019	Risankizumab and Cyclosporine	150 mg s.c. every 12 wk Cyclosporin: 200-300 mg daily	Partial response	Hypertension, creatinine increase	8
08/2019-03/2021	Guselkumab and Cyclosporine	Induction: 100 mg s.c. wk 0 and 4 Maintenance: 100 mg s.c. every 8 wk Cyclosporin: 250-350 mg daily	No repsonse		8
03/2021-12/2021	Brodalumab and Cyclosporine	Induction: 210 mg s.c. wk 0, 1, and 2 Maintenance: 210 mg s.c. every 2 wk	No response		10
12/2021-02/2022	Bimekizumab	Induction: 320 mg s.c. wk 0, 4, and 8	Partial response	Genital candidiasis	8
03/2022-05/2022	Upadacitinib	15-30 mg daily	No response		9
12/2022-03/2023	Tildrakizumab	Induction: 100 mg s.c. wk 0	No response	First onset of GPP 4 wk after application of tildrakizumab	14
03/2023-01/2025	Bimekizumab	Induction: 320 mg s.c. wk 0, 4, 8, 12, and 16 Maintenance: 320 mg every 8 wk	Partial response		10
01/2025-today	Spesolimab	Induction: 900 mg i.v. wk 0, 2, 6, and 10 Maintenance: 300 mg s.c. every 4 wk	Response	Telogen effluvium	4

*ACH*, Acrodermatitis continua of Hallopeau; *BW*, body weight; *DLQI*, Dermatologic Life Quality Index; *GPP*, generalized pustular psoriasis; *i.v.*, intravenous; *NA*, not available; *PUVA*, psoralen and ultraviolet a therapy; *s.c.*, subcutaneous.y; *s.c.*, subcutaneous.

In January 2025, with a body mass index of 35.3 kg/m^2^, the patient presented with multiple pustules (Fig 1, *A* and *B*), a Generalized Pustular Psoriasis Area and Severity Index of 0 (range 0-72), and a Pustular Psoriasis Area and Severity Index of 8.8 (range 0-72). The modified Nail Psoriasis Severity Index and the static Physician Global Assessment for ACH were 32 (range 0-64) and 4 (range 0-4), respectively. Laboratory investigations, including complete blood count, liver function tests, and C-reactive protein, were within normal limits.

**Fig 2:**
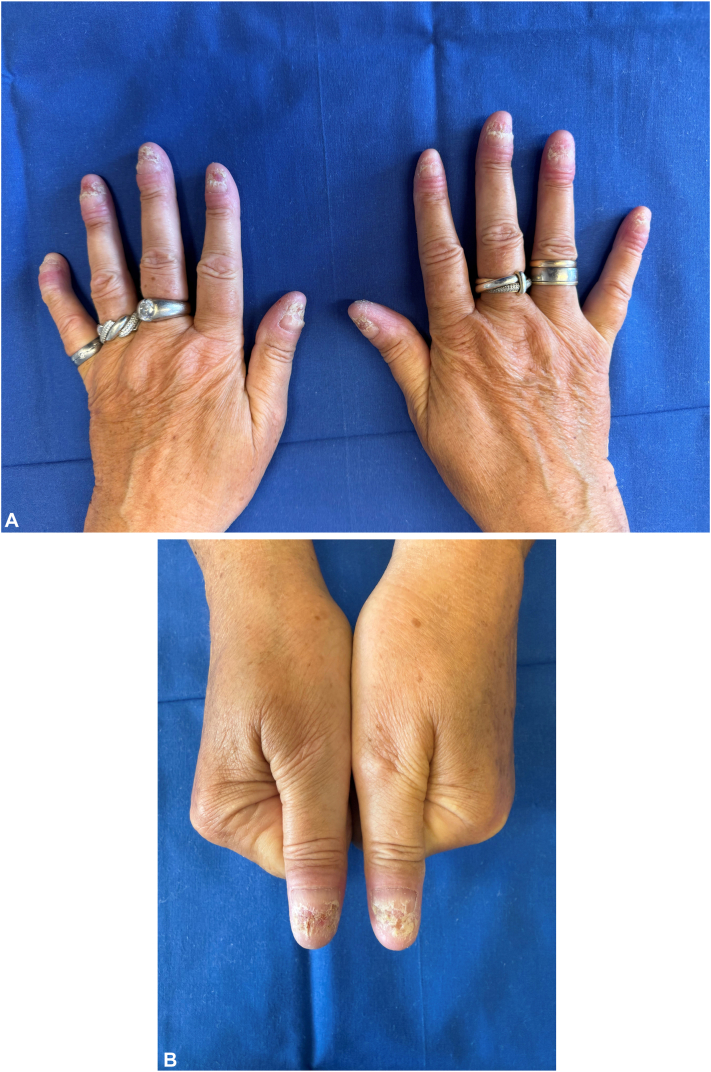
**A,** Patient’s hands: acrodermatitis continua of Hallopeau after starting spesolimab therapy. **B,** Patients thumbs: acrodermatitis continua of Hallopeau after starting spesolimab therapy.

Following the approval of an individualized off-label treatment by the statutory health insurance, the patient received a single dose of 900 mg of spesolimab intravenously in January 2025. Given a partial but transient response, 3 additional intravenous doses were administered (weeks 2, 6, and 10). Telogen effluvium occurred after the third dose and was managed with 5% topical minoxidil applied once daily. The patient’s cutaneous symptoms improved substantially (Fig 2, *A* and *B*). Dermatologic Life Quality Index score decreased to 6 (range 0-30), modified Nail Psoriasis Severity Index to 8 (range 0-64), and Pustular Psoriasis Area and Severity Index to 0.8 (range 0-72). Maintenance therapy with 300 mg subcutaneous spesolimab every 4 weeks was initiated, with ongoing clinical remission reported through July 2025. Notably, she also reported significant improvement in joint pain and morning stiffness beginning approximately 5 weeks after initial treatment.

## Discussion

This case clearly demonstrates the significant therapeutic challenges and unmet needs in managing refractory ACH, especially when it is associated with PsA and extensive nail disease. Spesolimab was approved in the United States (Food and Drug Administration) and Europe (European Medicines Agency) in 2022 for use in the treatment of generalized pustular psoriasis (GPP) flares in adults. In Germany, the Federal Joint Committee (Gemeinsamer Bundesausschuss) concluded that the additional therapeutic benefit of spesolimab compared to the chosen comparator was not sufficiently proven. This assessment was based primarily on the EFFISAYIL 1 study,^6^ which the Gemeinsamer Bundesausschuss rated inadequate due to its short comparative analysis duration (8 days) and the appropriateness of treatment discontinuation prior to flare onset. While spesolimab is approved in the European Union and has undergone evaluation by the European Medicines Agency, it has been retracted from the German market and now necessitates supply from other EU countries.

Wen et al^7^ reported a case of a patient with ACH and GPP, noting a rapid regression of both skin and nail lesions. To date, there have been reports of 2 patients treated with spesolimab solely for ACH. Treatment effectiveness has been described in 3 other patients.^8^, ^9^, ^10^ Currently maintenance dosing for ACH is not yet defined. A significant aspect of the patient's treatment plan involved a transition from intravenous spesolimab to subcutaneous administration. This change was carefully considered to align with the prevention of flare-ups of ACH, given that there are no specific data available for its prophylaxis. We therefore based our approach on the dosing guidelines established for GPP prevention.^4^ While hair loss associated with spesolimab is not listed as a side effect in the drug information, it warrants acknowledgment as a potential newly discovered side effect.

To the best of our knowledge, our report is the first to document both sustained remission and transition to subcutaneous spesolimab for maintenance.

Our observations suggest that spesolimab may exert broader immunological effects beyond pustular resolution, improving nail involvement and joint symptoms as well. Although telogen effluvium has not been documented as an adverse event, clinicians should remain vigilant. Further studies are needed to explore optimal dosing, long-term safety, and efficacy in both skin and joint manifestations of ACH.

## Conflicts of interest

Dr Augustin has served as a consultant, lecturer, or researcher and/or has received research grants from companies manufacturing drugs for psoriasis, including AbbVie, Almirall, Amgen, Biogen, BMS, Boehringer Ingelheim, Celgene, Centocor, Eli Lilly, Galderma, Hexal, Incyte, Janssen, Klinge, LEO, Medac, MSD, Mylan B.V., Novartis, Pfizer, Sandoz, Takeda, UCB, and Viatris. Dr Wagner has received payments/honoraria for lectures and presentations and/or received grants and/or participated in clinical trials from the following companies: AbbVie, AstraZeneca, Almirall, Beiersdorf, Boehringer-Ingelheim, Celltrion, Incyte, Johnson & Johnson, LEO Pharma, Lilly, Novartis, Moonlake, Sanofi, and UCB. Dr Stephan has received payments/honoraria for lectures and presentations and/or received grants and/or participated in clinical trials from the following companies: AbbVie, Almirall Hermal, Amgen, Beiersdorf, Boehringer Ingelheim, Bristol-Myers Squibb, Celgene, Celltrion, GlaxoSmithKline, Janssen-Cilag, LEO Pharma, Lilly, Medac, Novartis, Pierre Fabre, Sanofi Aventis, and UCB. Dr Grote received speaker fees from BMS and MSG and travel grants from Almirall, Sanofi, Celltrion, and Lilly. Dr Zirkenbach has no conflicts of interest to declare.
